# A Mathematical Model of a Midbrain Dopamine Neuron Identifies Two Slow Variables Likely Responsible for Bursts Evoked by SK Channel Antagonists and Terminated by Depolarization Block

**DOI:** 10.1186/s13408-015-0017-6

**Published:** 2015-02-27

**Authors:** Na Yu, Carmen C. Canavier

**Affiliations:** Department of Cell Biology and Anatomy, Louisiana State University School of Medicine, New Orleans, LA 70112 USA; Department of Mathematics and Computer Science, Lawrence Technological University, 21000 West 10 Mile Road, Southfield, MI 48075 USA

## Abstract

Midbrain dopamine neurons exhibit a novel type of bursting that we call “inverted square wave bursting” when exposed to Ca^2+^-activated small conductance (SK) K^+^ channel blockers in vitro. This type of bursting has three phases: hyperpolarized silence, spiking, and depolarization block. We find that two slow variables are required for this type of bursting, and we show that the three-dimensional bifurcation diagram for inverted square wave bursting is a folded surface with upper (depolarized) and lower (hyperpolarized) branches. The activation of the L-type Ca^2+^ channel largely supports the separation between these branches. Spiking is initiated at a saddle node on an invariant circle bifurcation at the folded edge of the lower branch and the trajectory spirals around the unstable fixed points on the upper branch. Spiking is terminated at a supercritical Hopf bifurcation, but the trajectory remains on the upper branch until it hits a saddle node on the upper folded edge and drops to the lower branch. The two slow variables contribute as follows. A second, slow component of sodium channel inactivation is largely responsible for the initiation and termination of spiking. The slow activation of the ether-a-go-go-related (ERG) K^+^ current is largely responsible for termination of the depolarized plateau. The mechanisms and slow processes identified herein may contribute to bursting as well as entry into and recovery from the depolarization block to different degrees in different subpopulations of dopamine neurons in vivo.

## Introduction

The activity of midbrain dopamine neurons, as reflected in levels of extracellular dopamine concentration and the fMRI BOLD signals in their target areas, is hypothesized to represent a reward prediction error [1] or, alternatively, confidence in a prediction of a desired outcome [2]. The firing pattern of dopamine neurons affects dopaminergic signaling; for example, electrical stimulation of dopaminergic cells at 40 Hz is much more effective in elevating dopamine extracellular concentration in rat striatum [3] than the same number of stimuli applied at 10 Hz. Dopamine (DA) neurons are regular pacemakers at 1–7 Hz in vitro [4, 5], but in vivo exhibit different firing patterns, including regular single-spiking, irregular single-spiking and burst firing both in freely moving [6] and anesthetized [7] rats. In the dopamine neuron literature, a single burst is operationally defined [8] as beginning with an ISI of less than 80 ms and terminating with an ISI of 160 ms. Bursts are often interspersed within episodes of single-spike firing [6], although at least some examples of rhythmic bursting have been observed in vivo [9]. Bursts are associated in awake animals with reward-related stimuli [10, 11], and they are referred to as a phasic signal in contrast to the tonic signal mediated by single-spike firing.

The origin of the operational definition of bursting is that DA neurons typically fire at 3–8 Hz [12] in the tonic, single-spike mode in vivo, so the operational criterion was developed to detect an episode of faster than normal firing. In contrast, in the mathematical neuroscience literature [13–15], bursting is often defined as a rhythmic alternation of spiking and quiescent episodes without reference to specific frequencies. We propose that although special dynamic mechanisms for bursting are not required to achieve a temporary acceleration in frequency, the intrinsic currents that characterize dopamine neurons provide burst mechanisms that may be harnessed as needed to facilitate single or multiple bursts. In many mathematical models of burst firing, there is an underlying slow oscillation in membrane potential, and bursts of spikes occur during the depolarized portion of the slow envelope, whereas the silent interburst interval is more hyperpolarized than the interspike intervals during a burst. This type of bursting is often called square wave bursting [13, 14]. Here, we will consider an anomalous type of bursting in which the silent, interburst interval occurs at more depolarized potentials than the average membrane potential observed during the interspike intervals within a burst.

Dopamine neurons exhibit multiple oscillatory modes under different conditions. For example, blocking spikes using TTX reveals an additional oscillation, an approximately sinusoidal, calcium-mediated slow oscillatory potential (SOP) [5, 16] that has a frequency in the spiking range. On the other hand, blocking (or negatively modulating) the small conductance SK potassium channel increases the tendency to burst rhythmically both in vivo [17, 18] and in vitro [5, 19, 20]. These bursts often terminate in depolarization block, similar to bursts in rats chronically treated with antipsychotic drugs [21]. Depolarization block is a state of silence at membrane potentials more depolarized than those that support the generation of action potentials, also called spikes. Clues to the burst mechanism are: (1) blocking both spikes and the SK channel induces underlying plateau potential oscillations [5, 22] in which the depolarized phase can last for seconds, and (2) L-type calcium channel agonists are also sufficient to elicit bursting whereas L-type calcium channel antagonists abolish bursting elicited by SK channel block [23]. We focus on bursts elicited in vitro by SK channel block that terminate in depolarization block. To our knowledge, our recent modeling study [24] is the only model that captures the curious nature of these SK channel block mediated bursts in dopamine neurons in which the most prominent silent phase is more depolarized than the membrane potential during the interspike intervals within the burst. Here we perform a bifurcation analysis of a slightly modified version of that model to elucidate the mechanism in a reduced single-compartment model, and then we show that it is viable in a morphologically realistic model as well.

## Methods

### Model Equations

**Fig. 1:**
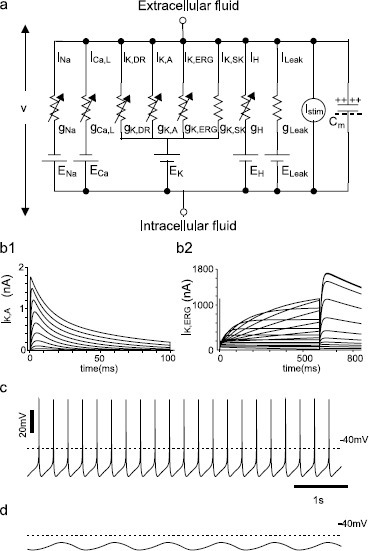
Calibration of dopamine neuron model. **a** The equivalent circuit for the conductance-based model with nonlinear conductance in parallel with the membrane capacitance $C_{\mathrm{m}}$. The maximal conductance and reversal potential of each current are indicated by $g_{x}$ and $E_{x}$, respectively. *The arrows* indicate time and voltage-dependent conductances. **b** Calibration of model K^+^ currents. **b1** The parameters of the description of the A-type K^+^ current were adjusted to fit published voltage clamp data from nucleated membrane patches from SNc dopamine neurons (representative current traces from Fig. 11A3 of [28]). The conductance used for these simulations (120 μS/cm^2^) was chosen to match the amplitude of the currents from the voltage clamp data, obtained with 100 ms steps from a holding potential of −100 mV to 50 mV in increments of 10 mV. **b2** The parameters of the description of the ERG-type K^+^ current were adjusted to fit published voltage clamp data (Fig. 1A of [32]) from human channels heterologously expressed in Xenopus oocytes. The conductance used for these simulations was chosen to match the amplitude of the currents from the voltage clamp data, obtained with 600 ms steps from a holding potential of −80 mV to −100 to 50 mV in increments of 10 mV. Tail currents were measured at −70 mV. **c** The model neuron exhibits slow pacemaker firing at 3.6 Hz under control conditions. **d** With $g_{\mathrm{Na}}$ set to zero and $I_{\mathrm{stim}}$ set to 35 pA, the model exhibits a Ca^2+^-dependent sinusoidal slow oscillatory potential (SOP)

The model (Fig. 1a) consists of a fast spiking sodium current ($I_{\mathrm{Na}}$) [25, 26], an L-type calcium current ($I_{\mathrm{Ca},\mathrm {L}}$) [27], a delayed rectifier ($I_{\mathrm{K},\mathrm{DR}}$) [28], a transient outward potassium current ($I_{ \mathrm{K}, \mathrm{A}}$) [28], an ether-a-go-go-related potassium current ($I_{\mathrm{K}, \mathrm{ERG}}$) [29], a calcium-activated small conductance SK potassium current ($I_{\mathrm{K},\mathrm{SK}}$), a nonspecific hyperpolarization-activated cation current ($I_{\mathrm{H}}$), and a leak current ($I_{\mathrm{Leak}}$) that is comprised of a nonspecific ($I_{\mathrm{L},\mathrm{NS}}$) and calcium ion specific component ($I_{\mathrm{L},\mathrm{Ca}}$). A small applied stimulus current $I_{\mathrm{stim}}$ was required for one simulation, and converted from pA to intensive units using the diameter d=15μm and L=25μm of the cylindrical somatic compartment. The conductances for these currents ($g_{i}$) are in parallel with the membrane capacitance $C_{m}=1\;{\text{μF/cm}}^{2}$. The differential equations for transmembrane potential are as follows:

$$C_{\mathrm{m}} \frac{dv}{dt} =- I_{\mathrm{Na}} - I_{\mathrm{Ca},\mathrm{L}} - I_{\mathrm{K},\mathrm{DR}} - I_{\mathrm{K},\mathrm{A}} - I_{\mathrm{K},\mathrm {ERG}} - I_{\mathrm{K},\mathrm{SK}} - I_{\mathrm{H}} - I_{\mathrm{Leak}} + 0.1 I_{\mathrm{stim}} /\pi\, dL. $$

Maximal conductances in μS/cm^2^ were $g_{\mathrm{Na}}=6000$, $g_{\mathrm{Ca},\mathrm{L}}=139$, $g_{\mathrm{K},\mathrm{DR}}=1117$, $g_{\mathrm{K},\mathrm{A}}=1680$, $g_{\mathrm {K},\mathrm{ERG}}=130$, $g_{\mathrm{K},\mathrm{SK}}=70$, $g_{ \mathrm{L},\mathrm{NS}}= 280$, $g_{ \mathrm{L},\mathrm{Ca}}=2.45$, $g_{\mathrm{H}}=78$.

The sodium current description, $I_{\mathrm{Na}} = g_{\mathrm{Na}} m^{3} h h_{s} ( v-60 )$, was modified from that given by Ji et al. [20] by making the slopes of the half-activation of *m* and fast inactivation of *h* slightly less steep. We also modified the slow component of sodium channel inactivation $h_{s}$ to better fit the data from [26] as explained in our earlier study [24]. The L-type current description, $I_{\mathrm{Ca},\mathrm{L}} = g_{\mathrm{Ca},\mathrm{L}} l ( v-50 )$, is similar to those used in our previous models [20, 24, 30] and represents only the fraction of calcium current activated near the spike threshold. The delayed rectifier description, $I_{\mathrm{K},\mathrm{DR}} = g_{\mathrm{K},\mathrm{DR}} n^{3} (v+90)$, was slightly modified (specifically the mathematical form of the time constant) from [20]. The description of the A-type potassium current, $I_{\mathrm{K},\mathrm{A}} = g_{\mathrm{K},\mathrm{A}} p (q_{1} /2 + q_{2} /2) (v+90)$, was fit to published voltage clamp data (Figs. 1b1 and b2) [28] using two components of inactivation, $q_{1}$ and $q_{2}$. The description of the H current, $I_{\mathrm{H}} = g_{ \mathrm{H}} m_{\mathrm{H}} (v+29)$, is very similar to that in [31]. The gating variables *m*, *h*, $h_{s}$, *l*, *n*, *p*, $q_{1}$, $q_{2}$ and $m_{\mathrm{H}}$ obey equations of the form $dx/dt= ( x_{\infty} -x)/ \tau_{x}$, with $x_{\infty} =1/[1+ \exp (- \frac{v- x_{\mathrm{half}}}{x_{k}} )]$, with parameters as given in Table 1.

**Table 1:** **Time and voltage dependence of model gating variables**

*x*	$x_{\mathrm{half}}$ (mV)	$x_{k}$ (mV)	Time constants: $\tau_{x}$ (ms)
*m*	−30.09	13.2	$0.01+ \frac{1}{a + b}$, *a* = −(15.6504 + 0.4043*v*)/[exp(−19.565 − 0.50542*v*)−1], *b* = 3.0212exp(−7.463e − 3*v*)
*h*	−54	−12.8	$0.4+ \frac{1}{a+b}$, *a* = 5.0754e − 4exp(−6.3213e − 2*v*), *b* = 9.7529exp(0.13442*v*)
$h_{s}$	−54.8	−1.57	20 + 580/(1 + exp(*v*))
*n*	−25	12	$\frac{22.7165}{1+ \exp (- (v+61.1253)/4.4429 )} [ \frac{1}{1+ \exp ( (v+36.8869) /9.7083 )} +0.0052]+0.7397$
*l*	−45	7.5	$1/\{ \frac{-0.020876 ( v+39.726 )}{\exp [ - (v+39.726)/4.711 ] -1} +0.19444 \exp [-(v+15.338)/224.21]\}$
$m_{\mathrm{H}}$	−77.6	17.317	26.21 + 3136/[1 + exp(−(*v* + 22.686)/29.597)]
*p*	−35.1	13.4	$\frac{95.5813}{1+ \exp (- (v+71.5402)/26.0594 )} [ \frac{1}{1+ \exp ( (v+62.5026)/6.5199 )} -0.5108]+48.2438$
$q_{1}$	−80	−6	6.1exp(0.015*v*)
$q_{2}$	−80	−6	$294.0087+[ \frac{55.8321}{ 1+ \exp ((v+52.5933)/4.9104 )} -5.2348][ \frac{1}{ 1+ \exp ((v-84.8594)/35.3239 )} ]$

The ERG potassium current uses a kinetic scheme described previously [19] in which transitions between the closed and inactivated state must pass through an open state,

$$c\;\begin{array}{c} \underset{\to }{\alpha_{o}}\\ \overset{\leftarrow }{\beta_{o}} \end{array}\;o\;\begin{array}{c} \underset{\to }{\alpha_{i}}\\ \overset{\leftarrow }{\beta_{i}} \end{array}\;i,$$

where *c*, *o* and *i* denote the fraction of ERG channels in the closed, open and inactivated states, respectively; and $\alpha_{o}$, $\beta_{o}$, $\alpha_{i}$ and $\beta_{i}$ are the voltage-dependent reaction rates. The transition rates between the closed and open states ($\alpha_{o}$ and $\beta_{o}$) are much slower than the transition rates between open and inactivated states ($\alpha_{i}$ and $\beta_{i}$). The current description, $I_{\mathrm{K},\mathrm{ERG}} = g_{\mathrm{K},\mathrm{ERG}} o (v+90)$, requires two differential equations:

$$\begin{aligned} \frac{do}{dt} =& \alpha_{o} ( 1-o-i ) + \beta_{i} i-o ( \alpha_{i} + \beta_{o} ), \\ \frac{di}{dt} =& \alpha_{i} o- \beta_{i} i \end{aligned}$$

with $\alpha_{o} =0.0036 \exp ( 0.0759 v )$, $\beta_{o} =1.2523 \mathrm{e}{-}5 \exp ( -0.0671 v )$, $\alpha_{i} =91.11\times \exp ( 0.1189 v )$ and $\beta_{i} =12.6 \exp ( 0.0733 v )$. The values resulted from our fit (Fig. 1b2) to previously published data [32].

The leak current description was separated into two components: $I_{ \mathrm{Leak}} = I_{\mathrm{L},\mathrm{Ca}} + I_{\mathrm{L},\mathrm{NS}}$, with a calcium component, $I_{ \mathrm{L},\mathrm{Ca}} = g_{ \mathrm{L},\mathrm{Ca}} (v-50 )$, and a nonspecific component, $I _{\mathrm{L},\mathrm{NS}} = g_{ \mathrm{L},\mathrm{NS}} (v+65) $, in order to keep track of Ca^2+^ ions separately for the material balance on free cytosolic Ca^2+^. The Ca^2+^ balance was required to determine the level of Ca^2+^ concentration [Ca] that activates the SK potassium current. The description for this current was taken from our previous papers [30]: $I_{\mathrm{K},\mathrm{SK}} = g_{\mathrm{K},\mathrm{SK}} ( v+90 ) /(1+ (0.00019/ [ \mathrm{Ca} ] )^{4} )$. The calcium balance is given by $\frac{d [ \mathrm{Ca} ]}{dt} = -2 f_{\mathrm{Ca}} ( I_{\mathrm{L},\mathrm{Ca}} + I_{\mathrm{Ca},\mathrm{p}} + I_{\mathrm{Ca},\mathrm{L}} )/(F d)$ where $[\mathrm{Ca}]$ is the Ca^2+^ concentration in mM, $f_{\mathrm{Ca}}= 0.018$ is the fraction of unbuffered free calcium, *d* is the diameter of soma (or of a compartment in the compartmental model) and *F* is Faraday’s constant. Extrusion of Ca^2+^ is modeled using a nonelectrogenic pump $I_{\mathrm{Ca},\mathrm{p}}$: $I_{\mathrm{Ca},\mathrm{p}} = I_{\mathrm {Ca},\mathrm{p},\mathrm{max}} /(1+[\mathrm{Ca}]/0.00055)$, with $I_{\mathrm{Ca},p,\max}=11\;{\text{μA/cm}}^{2}$.

### Full Morphology Model

In order to verify that the single-compartment model captured the features of the dendritic architecture of a real DA neuron, the model parameters established for single-compartment model were applied to a multi-compartmental model that was based on the reconstructed morphology [33] of an actual SNc DA neuron, except $f_{\mathrm{Ca}}$ was set to 0.0018. It consisted of a total of 41 compartments, including three somatic, and 38 dendritic compartments. The morphology description was obtained from the file labeled Nigra2a955-1 at NeuroMorpho.Org [34]. The equations were identical in all compartments to the equations given above for the single-compartment model except that the length and diameters of the compartments were variable, $I_{\mathrm{stim}}$ is applied only in the soma, and axial currents flowing between compartments were computed using an axial resistivity of 100 Ω-cm. For simplicity, all conductance densities were modeled as homogeneous throughout the somatodendritic tree, since dopaminergic dendrites are active [35]. However, we note that recent evidence [36] that the distal dendrites contribute less to setting the pacemaking frequency than the proximal ones implies a degree of heterogeneity that may be considered in future models.

### Simulation Methods

Numerical simulations for the single-compartment model were performed using code written in MatLab (MathWorks), whereas simulations for the multi-compartmental model based on the morphological reconstruction were performed using the simulation package NEURON [37, 38]. The bifurcation diagrams for the slow plateau potential were calculated with XPPAUT [39], whereas the bifurcation diagrams for bursting were generated by the MATCONT package [40].

## Results

### Single-compartment Model

The single-compartment model parameters were calibrated so that the model exhibits spontaneous pacemaking activity at about 3 Hz (3.6 Hz, Fig. 1c) under control conditions with all parameters set to their default value. We also confirmed that a calcium-driven, approximately sinusoidal, slow oscillatory potentials (SOP) can be obtained when bath application of TTX is simulated by setting $g_{\mathrm{Na}} =0$ (Fig. 1d) to block spiking. A small bias current of 35 pA was required in Fig. 1d to reveal this oscillation; although some dopamine neurons produce the SOP spontaneously, others require a small bias current [16]).

**Fig. 2:**
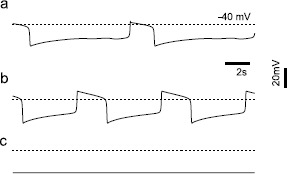
Oscillatory plateau potentials. **a** With $g_{\mathrm {Na}}$ and $g_{\mathrm{K},\mathrm{SK}}$ set to zero and all other parameters set to their default values, the model produces oscillatory plateau potentials of long duration. **b** Oscillatory plateau potentials persist when $g_{\mathrm {K},\mathrm{DR}}$ is also set to zero. **c** Oscillatory plateau potentials are abolished when $g_{\mathrm{Ca},\mathrm{L}}$ is also set to zero

Next we examined the ability of SK channel block to evoke oscillatory plateau potentials that resemble a square wave. With both $g_{\mathrm {Na}}$ and $g_{\mathrm{K},\mathrm{SK}}$ set to zero, the model produces depolarized plateaus lasting seconds, separated by periodic hyperpolarizing phases (Fig. 2a). These plateau potentials persist (Fig. 2b) when $g_{\mathrm{K},\mathrm{DR}}$ is set to zero to mimic bath application of TEA, consistent with experimental data [5]. Finally, we show that the plateau potentials in the model are abolished when $g _{\mathrm{Ca},\mathrm{L}}$ is also set to zero to mimic the application of nifedipine, again consistent with experimental data [22, 23].

### Model Reduction and Bifurcation Analysis (Without Spiking)

**Fig. 3:**
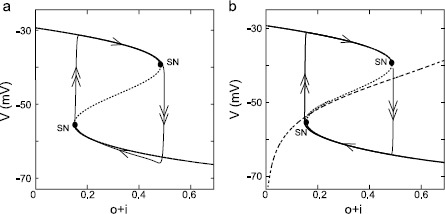
Fast-slow bifurcation diagrams for oscillatory plateau potentials. **a** Bifurcation analysis of the full model from Fig. 2b with $g_{\mathrm{Na}} = g_{\mathrm{K},\mathrm{SK}} =g_{\mathrm{K},\mathrm {DR}} =0$. *Solid* and *dotted lines* represent the stable and unstable fixed points on the bifurcation diagram, respectively. *Dots* indicate bifurcation points, with SN denoting saddle-node bifurcation. *Double* and *single arrows* indicate the direction of fast and slow changes in voltage, respectively, on *the closed curve* (*thin black lines*) that represents the limit cycle trajectory. **b** Same as **a** except the bifurcation diagram is equivalent to the voltage nullcline in the reduced two-variable system, and the nullcline for the $o+i$ pool is shown (*dashed curve*)

A bifurcation analysis was performed on the single-compartment model from Fig. 2b with $g_{\mathrm{Na}}$, $g_{\mathrm{K},\mathrm{SK}}$, and $g_{\mathrm{K},\mathrm{DR}}$ set to zero. The remaining state variables were $(v, l, p, q_{1}, q_{2} , m_{ \mathrm{H}}, o, i)$. The trajectory (thin black closed curve with arrows) corresponding to Fig. 2b was replotted in Fig. 3a in the plane of membrane potential (*v*) and the fraction of ERG channels ($o+i$) that are not closed, but are in either the open or inactivated state. The choice of these coordinate axes was motivated by a previous modeling study [19] that suggested that the $o+i$ pool of ERG potassium channels was the appropriate slow variable for a fast/slow analysis. As noted in Sect. 2, the transition rates between the closed and open states ($\alpha_{o}$ and $\beta_{o}$) are much slower than the transition rates between open and inactivated states ($\alpha_{i}$ and $\beta_{i}$), so the inactivated channels remain approximately at steady state with respect to the open fraction as their sum ($o+i$) changes slowly.

In the bifurcation analysis $(v, l, p, q_{1}, q_{2} , m_{\mathrm{H}})$ was taken as the fast subsystem, and the $o+i$ pool was the control parameter representative of the slow system. Branches of stable fixed points are indicated by dark curves and unstable one by the dotted part of the curve. The bifurcation diagram in Fig. 3a displays a “Z” shape constructed by two stable equilibrium branches at the top and bottom, and one unstable equilibrium branch in the middle. The unstable branch is created by the positive feedback due to the L-type Ca^2+^ channel, which is an inward current that turns on with depolarization, causing more depolarization as a result. The stable and unstable branches are connected by saddle-node (SN) bifurcation, and the trajectory jumps between depolarized and hyperpolarized branches at these points. The trajectory forms a limit cycle by following stable equilibrium branches. Membrane potential changes slowly when following the stable branches (denoted by single arrows in Fig. 3a), because the transmission between activation and closing states of ERG channels are slow, but changes rapidly when switching (double arrows) between stable branches due to switching on or off of the L-type calcium channel current.

Based on Fig. 3a, we theorized that a two-dimensional reduced model would be adequate to capture the dynamics of the system in Fig. 2b. The fast gating variables for the L-type calcium, A-type potassium and H currents $(l, p, q_{1}, q_{2} , m_{\mathrm{H}})$ were set to their corresponding steady states as a function of *v*, and we set $i= \alpha_{i} o/ \beta_{i}$ as explained above. The reduced two-variable system is

$$\begin{aligned} C \frac{dv}{dt} =&- g_{\mathrm{K},\mathrm{ERG}} ( o+i ) / ( \alpha_{i} + \beta_{i} ) (v- E_{\mathrm{K}} )- I_{\mathrm{Ca},\mathrm{L}} - I_{\mathrm{K},\mathrm{A}} - I_{\mathrm{Leak}} - I_{\mathrm{H}}, \\ \frac{d(o+i)}{dt} =&\alpha_{o} \bigl[ 1- ( o+i ) \bigr] - \beta_{o} \beta_{i} (o+i)/ ( \alpha_{i} + \beta_{i} ). \end{aligned}$$

The phase plane analysis of the reduced system is shown in Fig. 3b, and consists of the solution trajectory of the two-dimensional system superimposed on the voltage nullcline. This nullcline is the same as the bifurcation diagram from Fig. 3a. The trajectory for the reduced system follows the voltage nullcline exactly, whereas the trajectory for the full system in Fig. 3a follows it only approximately, but the correspondence is close. Furthermore, the nullcline (dashed curve) for the $o+i$ pool intersects the voltage nullcline in its unstable branch, resulting in a limit cycle solution.

### Inverted Square Wave Bursting with Two Slow Variables

**Fig. 4:**
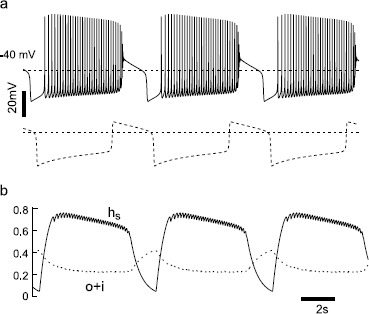
Inverted square wave bursting requires two slow variables. **a** *Top*: The model exhibits inverted square wave bursting with the default parameter set and $g_{\mathrm{K},\mathrm{SK}} =0$. *Bottom*: For comparison, the oscillatory plateau potentials from Fig. 2b (*dashed line*, with $g_{\mathrm{Na}}= g_{\mathrm{K},\mathrm{SK}} =g_{\mathrm{K},\mathrm {DR}} =0$) are shown. **b** The time course of slow sodium channel inactivation $h_{s}$ and $o+i$ pool of ERG channels that corresponds to the top trace in **a** is shown

Having clarified in the model the basis for the underlying plateau potential oscillations, we returned to the full model from Fig. 1c to simulate the curious type of bursting evoked by SK channel block in dopamine neurons. As stated in the Introduction, this type of bursting is curious because spiking in general occurs during the hyperpolarized phase of the underlying oscillation and quiescence occurs during the depolarized phase, which is inverted compared to square wave bursting [13, 14], so we use the term inverted square wave bursting to describe this pattern. Whereas under control conditions the model fires in a pacemaker fashion (Fig. 1c), when $g_{\mathrm{K},\mathrm{SK}}$ alone is set to zero to simulate block of the SK channels due to bath application of apamin, the model bursts (Fig. 4a). Bursting is characterized by three phases: spikes, depolarization block and hyperpolarized silence. For comparison, the oscillatory plateau potentials with both $g_{\mathrm{Na}}$ and $g_{\mathrm{K},\mathrm{SK}}$ set to zero is shown in dashed lines at the bottom of panel A, showing that the period, in this instance, is about the same with and without spikes.

Multiple time scales are inherent in bursting dynamics. We have identified two slow variables that are sufficient to generate this bursting pattern: the slow component of sodium channel inactivation $h_{s}$ and the $o+i$ pool of ERG potassium channels (Fig. 4b). Slow sodium channel inactivation terminates spike firing, whereas a slow increase in the pool of open ERG potassium channels terminates and repolarizes the plateau potential. Conversely, removal of slow sodium channel inactivation allows spiking to begin, and a slow decrease in the pool of open ERG potassium channels allows the membrane potential to enter the depolarized plateau supported by L-type calcium channel activation. Figure 4b illustrates the role of the slow variables in detail. Increasing depolarization during the silent phase triggers the start of the spiking phase. The frequency of spiking increases as the spiking phase progresses, and controls whether $h_{s}$ increases or decreases. Above a certain frequency, $h_{s}$ begins to decrease. When $h_{s}$ decreases below a critical level, there is insufficient sodium channel availability to support spiking, resulting in depolarization block. The other slow variable, the $o+i$ pool of ERG potassium channels, slowly decreases during spiking and slowly increases during depolarization block. When $o+i$ pool reaches a threshold value, the net current becomes outward, triggering a regenerative closing of the L-type Ca^2+^ channels and a sharp repolarization to the silent phase. During the silent phase, $h_{s}$ recovers from inactivation, and when $h_{s}$ recovers sufficiently, a new spiking phase is triggered.

### Model Reduction and Bifurcation Analysis (with Spiking)

**Fig. 5:**
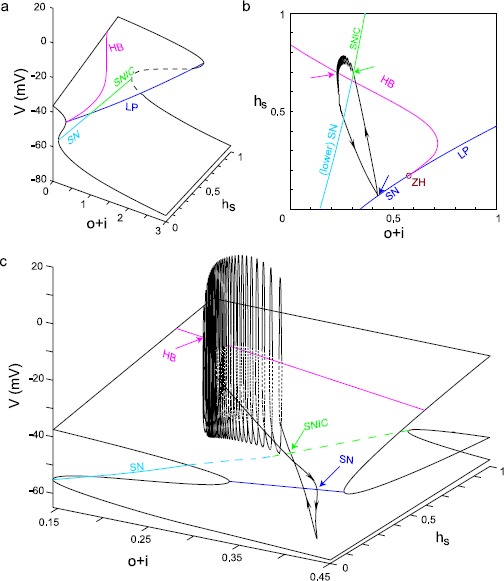
Bifurcation analysis for inverted square wave bursting (color online). **a** Three-dimensional bifurcation diagram with $h_{s}$ and $o+i$ pool as control parameters and *v* as the third variable. Three major bifurcation curves are demonstrated with different *colors* (*magenta*: Hopf bifurcation (HB), *blue*: limit points (LP), *green*: saddle node on an invariant cycle (SNIC), *cyan*: saddle node (SN)). The control parameter $o+i$ is expanded beyond its maximum of 1, in order to demonstrating the folding structure in 3D. *The dashed edge of the surface* indicates that it would not be visible if the surface were not transparent. **b** Two-parameter bifurcation diagram in the plane of $h_{s}$ and $o+i$ pool. The HB curve and LP curve merge at a zero-Hopf bifurcation (*red open circle*, denoted ZH). *The black curve* is the projection of bursting trajectory from the top of Fig. 4a in the same plane. *Arrows* indicate bifurcation points along the trajectory. **c** Rotated, expanded version of 3D bifurcation diagram from panel **a**. *The black curve* again indicates a 3-D version of the bursting trajectory

We then performed a bifurcation analysis of the system that produced the bursting in Fig. 4a (top), with $(v, m, h, l, n, p, q_{1}, q_{2} , m_{\mathrm{H}})$ as the fast subsystem, but in this case we used two control parameters, the $o+i$ pool as described in the bifurcation analysis without spiking, plus the slow component of sodium channel inactivation $h_{s}$. Figure 5a shows the numerically computed three-dimensional bifurcation structure with *v* as the third dimension, and gives the equilibrium points for which the derivatives of the fast subsystem, including that for the membrane potential, are zero. The fundamental Z-shape of the bifurcation curve for the lower-dimensional case in Fig. 4 that appears in the plane of *v* versus the $o+i$ pool, and is a result of the positive feedback loop mediated by $I_{\mathrm{Ca},\mathrm{L}}$, has been extended into a folding plane in Fig. 5a. The additional axis of $h_{s}$ allows a variable contribution of the regenerative sodium current to the positive feedback loop that creates the unstable branch of the Z; $I_{\mathrm{Na}}$ is also a depolarizing current that turns on with increasing depolarization. Note that the cross-sectional Z in the *v* versus the $o+i$ pool plane is much more pronounced for $h_{s} =1$ compared to $h_{s} =0$. The branch of lower saddle nodes (cyan curve, labeled SN) turns into a saddle node on an invariant circle branch (green curve, labeled SNIC) when the limit cycles initiated by the supercritical Hopf bifurcation (magenta curve, labeled HB) collide with the SNs. Here, we use the abbreviation SNIC to indicate a periodic orbit emerging from a homoclinic connection to a saddle node [41, 42].

The SN/SNIC branch constitutes an edge where the surface folds and is formed by joining all the saddle nodes on the lower branches of the Z in the 2D nullclines at each value of $h_{s}$. The presence of a supercritical Hopf bifurcation (magenta curve, labeled HB), introduced by the spiking currents, converts the branch of saddle nodes at the top of the Z into a limit point branch instead (blue curve, labeled LP). This is because, for most of its length in Fig. 5a, instead of being comprised of nodes between an unstable and a stable branch as in the 2D case, it is now mostly comprised of nodes between two unstable branches. This upper LP branch, like the lower SN branch, constitutes an edge where the surface folds back on itself; in contrast, the surface does not fold along the HB branch. The supercritical HB divides the top portion of the surface into stable (left side) and unstable (right side) regions. The middle (between the SN/SNIC branch and the LP branch) and bottom portions of the folding surface (below the SN/SNIC branch) are unstable and stable, respectively.

Figure 5b projects the three branches of bifurcation curves onto the plane of $h_{s}$ versus the $o+i$ pool. For clarity, we have omitted a number of neutral bifurcation curves and codimension-2 bifurcation points. The view has been expanded, as the $o+i$ axis has been expanded to cover the range of $[0, 1]$ instead of $[0, 3]$ as in Fig. 5a. In this view, it is evident that the HB curve collides with the LP curve at a zero-Hopf bifurcation (ZH, red open circle). Due to the stability of the adjacent upper surface, the portion of LP curve on the left side of ZH is also a saddle-node branch (limit point is a general term of which saddle node is a special case). The bursting limit cycle trajectory (black curve) is also projected on the $h_{s}$ versus $o+i$ pool plane. Since the curves have been collapsed into a single plane, not all apparent intersections in the plane actually occur. The bifurcation points along the trajectory are indicated by arrows. Spiking begins at the SNIC (green arrow) and terminates with a delay after passing through the supercritical Hopf (red arrow). The depolarized plateau terminates at a SN (blue arrow) and, as in the nonspiking case, quickly repolarizes as the L-type Ca^2+^ channel turns off regeneratively.

Figure 5c superimposes a three-dimensional version of the trajectory (black closed curve in Fig. 5b) from the simulation in Fig. 4a (top), which now includes membrane potential *v*, on a rotated version of the bifurcation diagram shown in Fig. 5a. The very first spike of burst spiking mode is initiated at the lower fold of the surface on a SNIC (green arrow, dashed SNIC line indicates this fold would be invisible if the upper lobe were not transparent). Spiking proceeds on the far side of the Hopf (magenta curve), in other words, behind it from this perspective. As $h_{s}$ decreases, the spiking mode terminates spikes through HB bifurcation (magenta arrow). The fast dynamic *v* trajectory causes the last spike to occur after the trajectory has crossed the HB point. Such “bifurcation delay” or “memory effect” has been reported in many studies (see for example [43–45]). The membrane potential remains on a depolarized plateau until sufficient channels accumulate in the $o+i$ pool to reach the upper SN bifurcation (blue arrow) and cause the trajectory to jump to the bottom stable lobe. Note that in this expanded and rotated view in Fig. 5c, only the SN portion of the LP curve is visible. The trajectory remains quiescent until $h_{s}$ recovers sufficiently to restart bursting spikes at the SNIC.

### Morphologically Realistic Model

**Fig. 6:**
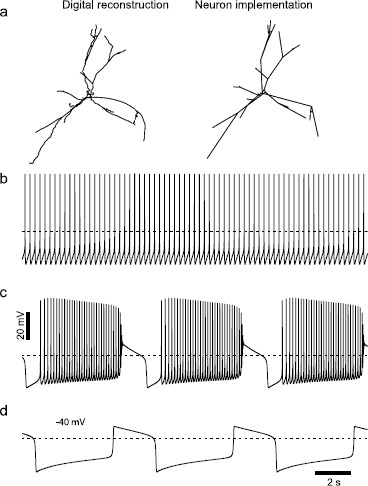
Simulation results of the NEURON model with a realistic morphology. **a** *Left*: digital reconstruction of neural morphology [33]. *Right*: Morphology rendered by the NEURON simulation package. **b** With the default parameter set except $f_{\mathrm{Ca}} =0.0018$, the model paces regularly at 3.5 Hz. Membrane potential from a somatic compartment is illustrated. **c** Setting $g_{\mathrm{K} , \mathrm{SK}}$ to zero produces inverted square wave bursting. **d** Setting $g_{\mathrm{Na}} = g_{\mathrm{K},\mathrm{SK}} =g_{\mathrm {K},\mathrm{DR}} =0$ produces oscillatory plateau potentials

We ported the same parameter set used in the previous sections to a realistic morphology (Fig. 6a, left) implemented using the NEURON simulation package (Fig. 6a, right) as described in the Methods. For simulations with no SK channel current, and therefore no dependence on the rate of calcium ion accumulation set by the diameter of each compartment, the exact same results were obtained for the single-compartment and the full morphology. Setting $g_{\mathrm{K},\mathrm{SK}}$ to zero in the multi-compartmental model resulted in bursting activity (Fig. 6c). In addition, the ability to generate oscillatory plateau potentials with $g_{\mathrm{Na}}$, $g_{\mathrm {K},\mathrm{SK}}$, and $g_{\mathrm{K},\mathrm{DR}}$ all set to zero was preserved (Fig. 6d). Therefore, in these cases, the bifurcation structure of this very complex multi-compartmental model, which is not always possible to determine directly, is qualitatively identical to the reduced, one-compartment model examined in the previous sections. However, for simulations with nonzero SK conductance (Fig. 6b), the parameter set from the single-compartment model resulted in quiescence rather than spiking. The dendrites have a higher surface to volume ratio than the single-compartment somatic model. This allows for faster accumulation and removal of free Ca^2+^, and faster activation of the SK channel current, which abolished pacemaking. In order to match the frequency and waveform of pacemaking in the single-compartment model, the free Ca^2+^ fraction $f_{\mathrm{Ca}}$ was reduced by a factor of 10, from 0.018 to 0.0018.

## Discussion

Here we describe the bifurcation structure of a type of bursting observed in midbrain dopamine neurons in vitro when exposed to SK K^+^ channel blockers. This type of bursting has three phases: hyperpolarized silence, spiking, and depolarization block. Two slow variables are required, and the three-dimensional bifurcation diagram is a folded surface with upper (depolarized) and lower (hyperpolarized) branches. The activation of the L-type Ca^2+^ channel largely supports the separation between these branches. Spiking is initiated at a SNIC at the edge of the lower branch and forms a limit cycle around the unstable fixed points on the upper branch. Spiking is terminated at a supercritical Hopf bifurcation, but the trajectory remains on the upper branch until it hits a saddle node and drops to the lower branch. The slow variables contribute as follows. The slow component of sodium channel inactivation is largely responsible for initiation and termination of spiking. The slow activation of the ERG K^+^ current is responsible for termination of the depolarized plateau.

### Relationship to Previous Bifurcation Analyses

The first dynamic classification of bursting neurons included three types of bursting [13, 14]: square wave, parabolic and elliptical. Of these, only parabolic bursting requires two slow variables, and bursting is initiated by a SNIC and terminated by a SNIC. More recent work exhaustively catalogued every possible bifurcation type leading to burst firing [15]. The bifurcation structure we presented here for inverted square wave bursting is most closely related to Izhikevich’s fold/Hopf bursting and circle/Hopf bursting (Figs. 63 and 67, respectively, in [15]). For all three cases, the surface on which the derivative of the membrane potential is equal to zero has the same fundamental structure: a “Z”-shape comprised of upper and lower surfaces connected at folds by an unstable surface corresponding to the middle branch of the “Z”.

In the circle/Hopf bursting as illustrated in Fig. 67 of [15], there are depolarized plateaus on the upper surface alternating with episodes of hyperpolarized silence on the lower surface. Transitions between these “up” and “down” states are caused by fold, or saddle node, bifurcations at the edges of the upper and lower surfaces. The circle (SNIC) and Hopf bifurcations that initiate and terminate spiking both occur on the upper surface, so the sequence of bifurcation starting with spike initiation is SNIC-HB-SN-SN, and there are nonspiking, silent periods before and after spiking on the upper surface, as well as during the entire trajectory on the lower surface. In the fold/Hopf bursting as illustrated in Fig. 63 of [15], spiking is initiated on the fold of the lower surface causing a jump to the upper surface and termination on the upper surface via a Hopf bifurcation, followed by a quiescent period of depolarization block, so the bifurcation scheme is SN-HB-SN.

In our model, spiking is also initiated on the edge of the lower fold, but at a SNIC (green arrow in Fig. 5c) instead of a SN, and terminates on the upper surface at a Hopf (magenta arrow). Then the trajectory remains on the upper surface until the edge is reached at a fold (SN, dark blue arrow) and the trajectory moves across the lower surface. The bifurcation structure, starting with spike initiation, is SNIC-HB-SN. Since the burst categorization given by Izhikevich is based on the bifurcations that initiate and terminate spiking, inverted square wave bursting is, by definition, a special case of the circle/Hopf bursting. The important distinction from the example given by Izhikevich is that, in our model, spiking appears to emerge smoothly from the “down” state, or hyperpolarized silence, which is consistent with the experimental phenomenon we are trying to model. In Izhikevich’s example of circle/Hopf bursting, the transition to the “up state” clearly occurs before spiking begins, which is not consistent with experimental observations in dopamine neurons.

Another important distinction is that in both the circle/Hopf and the fold/Hopf examples given by Izhikevich, the depth of the spike after-hyperpolarization is limited by the unstable part of the surface corresponding to the middle branch of the “Z” shape, because the spiking trajectory spirals around unstable fixed points on the upper surface above the unstable branch. The second slow variable allows for the trajectory to move between the SNIC (green arrow in Fig. 5c) and the HB (magenta arrow) in a direction parallel to the lower fold, but in a region of state space beyond the extent of the lower surface, removing the limitation on the spike after-hyperpolarization. A bursting pattern similar to that in this study, in which spiking was also initiated smoothly from the silent hyperpolarized phase and terminated in depolarization block, was previously identified in a model of pancreatic beta cell activity [46]. That study also found that two slow variables are necessary to construct the folded bifurcation plane on which these complicated bursting trajectories arise.

In Fig. 3, we show that the transitions into and out of the depolarized phase of the oscillatory plateau potentials with both $g_{\mathrm {K},\mathrm{SK}}$ and $g_{\mathrm{Na}}$ set to zero occur at a saddle node, in agreement with a previous bifurcation analysis [17] of the same oscillatory phenomenon in a similar model of a dopamine neuron. However, in that study, only one slow variable was used and removing the sodium channel block produced “bursts” in which a single spike rides a depolarizing wave, rather than inverted square wave bursting.

### Relationship to Previous Models of Bursting in Dopamine Neurons

This paper builds on previous modeling results from our group and others. The first models of bursting in dopamine neurons [47, 48] focused on the ability of the NMDA receptor current ($I_{\mathrm{NMDA}}$) to support depolarization underlying bursting, and the ability of the electrogenic sodium pump ($I_{\mathrm{Na},\mathrm{p} }$) to terminate bursts, a mechanism suggested by experiments performed in vitro [49] as well as the known contribution [50–52] of NMDA receptors to burst firing in vivo. One of the early models [47] postulated a role for the Ca^2+^-activated K^+^ conductance in burst termination. Although this current likely contributes to burst repolarization under some circumstances, blocking this current facilitates bursting, as described in the Introduction. The explanation for this counterintuitive experimental observation requires an understanding of the role of the SK current in allowing only one spike per Ca^2+^ channel-mediated depolarization [30, 53, 54].

A clue to the mechanism by which blocking the SK conductance facilitates bursting was provided by models [17, 55, 56] that showed that the Ca^2+^-mediated slow oscillation in membrane potential (SOP) observed when spikes are blocked with TTX is caused by the interplay between the L-type Ca^2+^ channels and the Ca^2+^-activated SK K^+^ channels. Moreover, decreasing the level of the SK conductance converted the sinusoidal oscillatory potential (similar to those in Fig. 1d) to oscillatory plateau potentials (similar to those in Figs. 2a and b). When the fast sodium conductance in not blocked, the sinusoidal oscillatory potentials support single spiking; in contrast, the oscillatory plateau potentials support bursting. The role of the ERG current in termination of plateau potentials was elucidated previously [19, 20], but the inverted square wave bursting that the SK block can induce was only recently modeled by our group [24]. Most recently, a type of bursting in which $I_{\mathrm{NMDA}}$ provides the depolarizing drive and the ATP-mediated K^+^ current provides the repolarizing drive was identified [57] in a subpopulation of dopamine neurons and modeled by our group [58]. The degree to which different burst mechanisms and currents contribute to bursting in distinct subpopulations of dopamine neurons [59, 60] in vivo is an open question.

### Implications for Dopaminergic Signaling

Here we have focused on (1) the ability of the SK K^+^ current to inhibit bursting, (2) the ability of the L-type Ca^2+^ channel to support depolarization underlying a burst and any subsequent depolarization block, (3) the role of the slow ERG K^+^ current in opposing the L-type Ca^2+^ channel to repolarize the membrane after an episode of depolarization block, and (4) the role of the slow component of sodium channel inactivation in initiating and terminating spiking. The role of the second, slow component of sodium channel inactivation $h_{s}$ in the induction of depolarization block in dopamine neurons has previously been suggested [20, 24, 61], but here was rigorously examined in the context of spontaneous bursting. The ability of dopamine neurons to enter into and recover from depolarization block may be physiologically significant because bursts terminating in depolarization block have been observed in vivo in rats chronically treated with an antipsychotic [21], and the therapeutic value of antipsychotics has been attributed to their ability to induce depolarization block in dopamine neurons [62]. One side effect of antipsychotic drugs is blocking the ERG K^+^ current [63]; since the ERG K^+^ current contributes to recovery from depolarization block, it is possible that this side effect contributes to therapeutic efficacy [63].

Although the simplified model in this paper relies exclusively on the ERG K^+^ current to repolarize the membrane after a plateau potential or the depolarization block that can follow a burst, it is likely that several slow outward currents, such as $I_{\mathrm{Na},\mathrm{p}}$ or additional slow K^+^ currents contribute to burst termination and recovery from depolarization block in vivo, because blocking the ERG K^+^ current only rarely causes cells to fail to repolarize from the plateau potential induced by negative modulation of the SK channel current [20].

During bursting induced by SK block in vitro, spiking begins at a low frequency, but just prior to entry into depolarization block, often a burst of fast frequency, full amplitude spikes is emitted; these particular spikes may provide an in vitro analog of operationally defined bursts (see Introduction) in vivo. Unfortunately, the model emits only a few, partial amplitude very fast frequency spikes upon entry into depolarization block (Fig. 4a, top). This is an aspect of the model that provides an opportunity for further improvement in our understanding of the dynamics and bifurcation structure underlying this type of bursting. The ability of the SK channel to modulate bursting is physiologically relevant because the level of SK current activation is regulated endogenously in dopamine neurons. For example, repeated ethanol exposure and withdrawal reduces the contribution of the SK conductance in VTA dopaminergic neurons, which increases their tendency to burst [64]. The bifurcation structure of bursting in dopamine neurons in the model critically depends on the currents $I_{\mathrm{K},\mathrm{SK}}$, $I_{\mathrm{L},\mathrm{Ca}}$, $I_{\mathrm{ERG}}$, and $I_{\mathrm{Na}}$, and this result likely generalizes to physiological dopamine neurons, with broad implications for therapeutic strategies for disorders such as schizophrenia and alcohol addiction.
